# Integrated geophysical prospecting for deep ore detection in the Yongxin gold mining area, Heilongjiang, China

**DOI:** 10.1038/s41598-025-92108-3

**Published:** 2025-03-01

**Authors:** Yechang Yin, Jun Chen, Zhonghai Zhao, Yuanjiang Yang, Chenglu Li, Haina Li, Xiang Zhao

**Affiliations:** 1 College of Mining, Liaoning Technical University, Fuxin, Liaoning, 123000 China; 2 Liaoning Key Laboratory of Green Development of Mineral Resources, LNTU, Fuxin, Liaoning, 123000 China; 3 Heilongjiang Institute of Natural Resources Survey, Harbin, Heilongjiang, 150036 China

**Keywords:** Geology, Geophysics

## Abstract

Geophysical exploration techniques play a pivotal role in enhancing the accuracy of mineral prospecting predictions. However, relying solely on individual methods often introduces uncertainties. This study presents a case study from the Yongxin gold deposit, where we integrated audio-frequency magnetotelluric (AMT) methods with gravimetric surveying and high-resolution magnetic profiling to overcome this challenge. Advanced three-dimensional modeling techniques were utilized to precisely delineate lithological variations and deep-seated mineralization features inherent to the area. The inversion and interpretation of cross-sectional AMT data provided insights into the subsurface structure down to a depth of 1.5 km. This enhanced data reliability was achieved through an integrated interpretation constrained by multiple datasets, enabling a more accurate inference of the deeper geological framework. Furthermore, by amalgamating various datasets, we uncovered characteristics of deep mineralization, the three-dimensional configuration of mineralization-related rock masses, and the spatial orientation of known ore deposits. This holistic approach facilitated a comprehensive understanding of the deeper geological formations. A detailed analysis of ore-controlling structures and exploration markers led to the development of a tailored geological-geophysical model for mineral exploration within the study area, serving as a valuable reference for future deep exploration efforts.

## Introduction

In recent years, with the gradual depletion of shallow mineral resources, the exploration and development of mineral resources at depths ranging from 500 to 2000 m have become the primary focus of ore deposit scientists^1,2^. In this context, traditional geological exploration methods face numerous limitations, and the discovery of new ore deposits has become increasingly challenging^3–6^. Therefore, the significance of high-precision, high-resolution, and easy-to-operate geophysical exploration techniques has become paramount^7,8^. Among these, the Audio-frequency Magnetotelluric (AMT) method has been widely utilized in deep mineral exploration due to its advantages such as high work efficiency, large exploration depth, ability to penetrate high-resistivity layers, and strong resolution for low-resistivity layers^9–13^.

Notably, integrated geophysical exploration methods have achieved remarkable results in various mining areas worldwide. For instance, Lahti et al. conducted a 2D smooth inversion of AMT data to visualize the subsurface conductivity structure of the Outokumpu belt in eastern Finland. This provided valuable insights for deep exploration and ore body positioning prediction^8^. Similarly, Wang et al., in the Jiaojia gold mineralization belt of Shandong, integrated geological, tectonic, drilling data, and controlled-source audio-frequency magnetotelluric (CSAMT) survey profiles to create a three-dimensional geological model. Through a comprehensive analysis of lithology, physical properties, and the forward interpretation of the geophysical field, they constructed a three-dimensional geological-geophysical model and identified six prospective mining targets^14^. Furthermore, Zhang et al. employed AMT technology to generate two-dimensional geoelectrical profiles in the Hongshishan research area of Gansu Province. By correlating surface lithology with physical properties like resistivity and magnetic susceptibility, they established a three-dimensional geological model of the ophiolite mélange zone. This model offers crucial insights into the occurrence and contact relationships, which are essential for exploring the formation mechanisms of basic and ultrabasic rocks^15^.

To more accurately delineate the ore body morphology and predict the deep mineralization potential, we employed an integrated geophysical exploration method primarily based on Audio-frequency Magnetotelluric (AMT) surveys, supplemented by gravity areal measurements and high-magnetic profile surveys in the Yongxin gold mining area. We deployed a total of 25 AMT profiles in the study area and processed the field data using advanced two-dimensional AMT inversion techniques. Through this approach, we obtained detailed electrical structure information up to a depth of 1.5 km below the surface. Furthermore, by integrating geological, physical, and gravimagnetic data from the study area, we conducted in-depth interpretation and constraint analysis of the anomalous bodies within the electrical structure, resulting in a more precise geological-geophysical model. This model not only reveals the deep distribution characteristics and relationships of various mineralization elements in the study area but also provides a strong exploration direction and deployment basis for subsequent identification of favorable mineralization zones and the establishment of ore-prospecting prediction models.

## Geological setting

### Regional geology

**Fig. 1:**
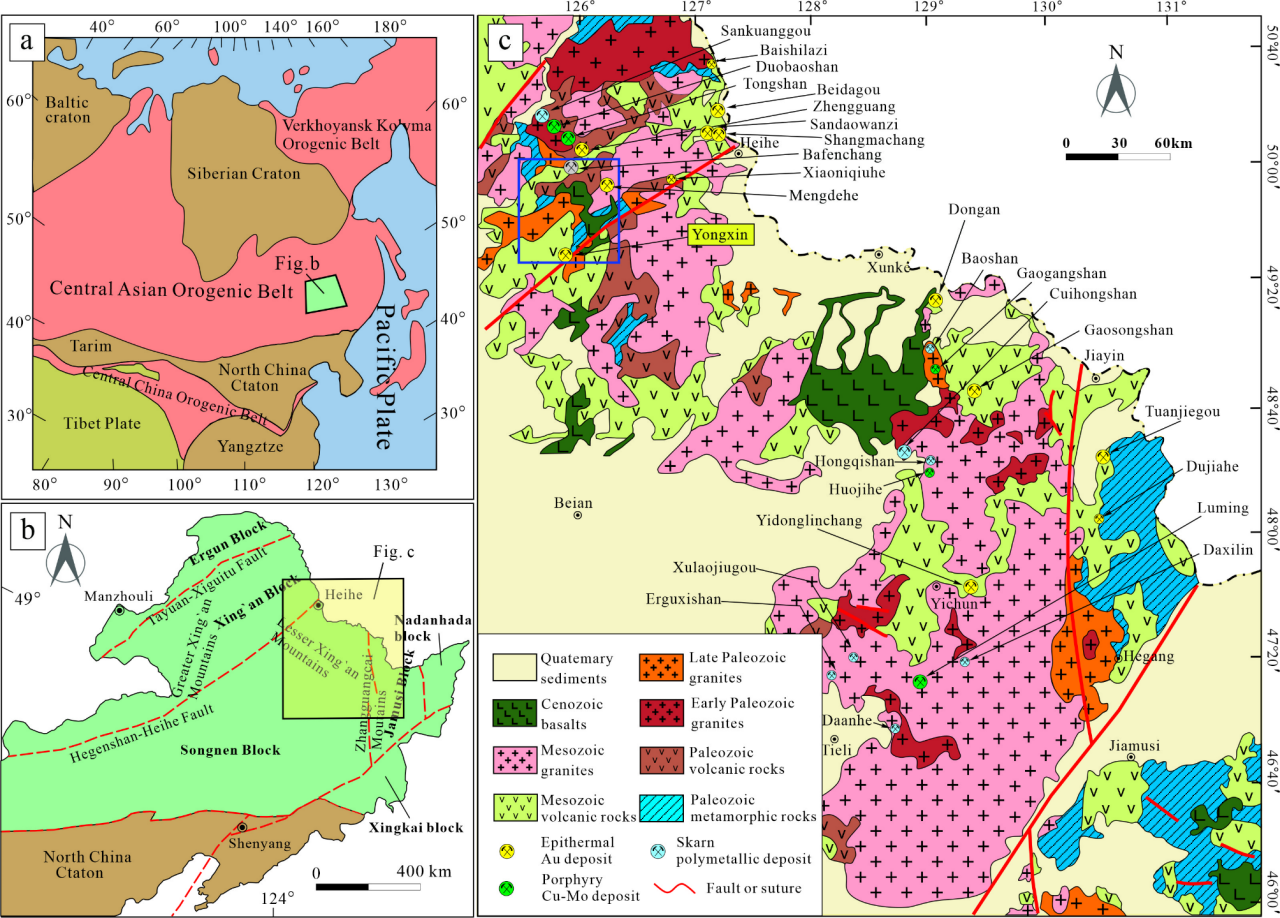
Geotectonic location map. (**a**) Simplified tectonic division of China and the surrounding area, modified after Liu et al.^30^. (**b**) Tectonic subdivisions of NE China, modified after Zhao et al.^31^. (**c**) Simplified geological map of the Lesser Xing’an Range, modified after Zhao et al.^32^ (The figure is generated using CorelDraw X9 (http://www.coreldraw.com/cn/) by Jun Chen).

The study area is situated in the eastern segment of the Central Asian Orogenic Belt (Fig. 1a), specifically at the tectonic interface of the Xing’an and Songnen blocks in the Inner Mongolia-Great Xing’an orogenic belt (Fig. 1b). This is a geologically complex region that has undergone prolonged tectonic and magmatic evolution^16,17^. Since the Paleozoic era, it has been influenced by the tectonic shifts between the Siberian and North China Plates, as well as the Paleo-Asian Ocean’s development and eventual closure^18–20^. During the Mesozoic, key events included the tectonic changes related to the Mongol-Okhotsk Ocean and the Pacific Plate’s subduction, followed by deep faulting in the Cenozoic^21^. These multiple tectonic and magmatic activities have resulted in a diverse geological profile, including Paleozoic volcanic and metamorphic rocks, Mesozoic continental volcanic rocks, Paleogene fluvial-lacustrine sedimentary rocks, and Phanerozoic granites^22,23^. This region of Northeast China is renowned for its intense and intricate tectonic and magmatic history. As a result, it hosts a wide range of precious and non-ferrous metal deposits, including copper-molybdenum, iron-copper (molybdenum) polymetallic, and epithermal gold deposits^24–29^ (Fig. 1c).

### Ore deposit geology

**Fig. 2:**
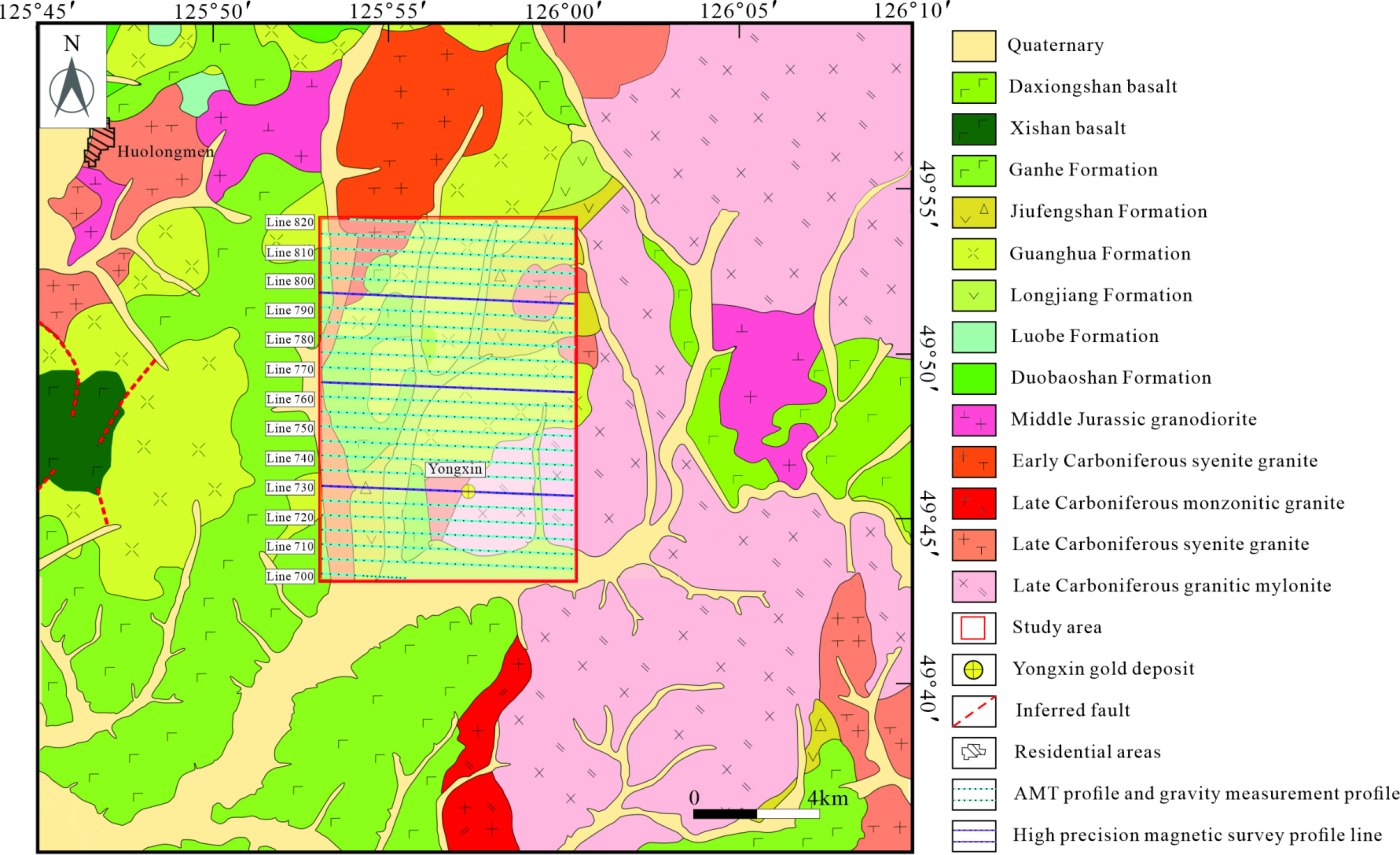
Geological map of the Yongxin gold deposit. (The figure is generated using CorelDraw X9 (http://www.coreldraw.com/cn/) by Jun Chen).

The Yongxin gold deposit, situated 70 km northeast of Nenjiang in Heilongjiang Province and 115 km southwest of Heihe City, boasts a substantial gold reserve of approximately 20 tonnes with an average grade of 4.10 g/t^33^. The mining area comprises various rock types, predominantly Late Carboniferous syenogranite, granitic mylonite, Early Cretaceous volcanic rocks, and other subvolcanic formations, with a minor outcrop of granodiorite (Fig. 2).

The Early Cretaceous volcanic rocks primarily comprise the Longjiang Formation, the Guanghua Formation, and the Ganhe Formation. The Longjiang Formation is distributed in the central and northern parts of the mining area, mainly consisting of intermediate-acidic volcanic rocks. The Guanghua Formation is primarily located in the northern part of the mining area, predominantly consisting of acidic volcanic rocks. The Ganhe Formation appears sporadically in the southwestern corner of the mining area, mainly composed of andesite, vesicle andesite basalt, and vesicular basalt. These Early Cretaceous volcanic rock eruptions unconformably overlie the mining area’s Syenogranite, granitic mylonite, and granodiorite. Early Cretaceous diorite-porphyrite and granite porphyry exhibit vein-shaped outputs oriented northeast to north-northeast, roughly parallel to the ore body.

The Yongxin deposit features two gold ore bodies controlled by a northeast-oriented fault zone, mainly found at the contact between Early Carboniferous syenogranite and mylonite^34^. These ore bodies extend in a northeast direction, with a gentle wave-like profile on cross-sections and structures aligned end-to-end. The main ore body, designated as No. 1, spans 375 m in length, with a maximum width of 73 m, a minimum width of 7 m, and an inclination angle ranging from 20° to 30°. It extends to a depth of approximately 800 m.

The Yongxin deposit is an epithermal gold deposit. The primary ore types are predominantly hydrothermal cemented breccia, quartz vein type, and altered rock type, with occasional small amounts of mylonite type. The ores exhibit various textures, including euhedral-hypidiomorphic granular, xenomorphic granular, brecciated, disseminated, veinlet, and vug or drusy. The primary metallic minerals are pyrite, galena, sphalerite, and minor chalcopyrite, with rare occurrences of native gold. Hematite and goethite are present as secondary minerals, while the lode minerals consist mainly of quartz, potassium feldspar, calcite, sericite, chlorite, epidote, and a small amount of adularia.

### Analytical methods

This study employed the V8 networked multi-functional electrical method system, developed by Phoenix Geophysics of Canada, to conduct audio-frequency magnetotelluric (AMT) surveys. The V8 system is capable of capturing natural electromagnetic fields within a frequency range of 0.35 Hz to 10,400 Hz, enabling deep subsurface exploration. The fieldwork involved three main stages: site selection, electrode deployment, and data acquisition.

*Site selection and electrode deployment*. The survey area covered approximately 100 km2 in the Yongxin gold mining region. A total of 25 east–west oriented survey lines were established, spaced 500 m apart, with measurement points positioned every 200 m along each line. This configuration yielded 1012 measurement points (Fig. 2). A cross-shaped electrode array was utilized, with an azimuth error margin of no more than 1° to ensure accurate orientation. The electrode spacing was set to 50 m for optimal signal resolution.

*Data acquisition*. A multi-station synchronous observation method was adopted, allowing simultaneous data collection at multiple measurement points^3^. Each station recorded both electric (Ex, Ey) and magnetic (Hx, Hy) field components. The sampling frequency was adjusted based on the target depth, with higher frequencies (e.g., 10,400 Hz) for shallow structures and lower frequencies (e.g., 0.35 Hz) for deeper exploration. The data acquisition time at each station was set to 2 h to ensure sufficient signal-to-noise ratio.

*Data processing and inversion*. Processed data were inverted in the Reservoir Geophysics Inversion System (RGIS), a comprehensive software package used for geophysical inversion and modeling. The inversion parameters included a starting model with a homogeneous half-space resistivity of 100 Ω m and a maximum iteration number of 100. The final resistivity models were validated through comparison with known geological structures and borehole data.The detailed data processing methods refer to the research conducted by Regean Pitiya et al.^35^.

*Quality control*. The resistivity measurements exhibited a relative error of ± 1.99% in the transverse electric (TE) mode and ± 2.37% in the transverse magnetic (TM) mode, demonstrating the high quality and reliability of the collected data. The consistency between the TE and TM modes further confirmed the robustness of the results^36^.

## Result

### Physical properties

This study collected rock samples and analyzed their electrical properties, density, and magnetic susceptibility, which reflect the physical characteristics of the rocks in the study area (Table 1). The findings reveal distinct variations in these properties among different rock types.

**Table 1:** Physical properties of rocks in the study area.

Type	Lithology	Resistivity (Ω m)		Magnetic susceptibility (10–5 SI)		Density (103 kg/m^3^)	
		Variation range	Average value	Variation range	Average value	Variation range	Average value
Intrusive rock	Middle Jurassic granodiorite	3435.4–14,977.9	5178.4	80.0–357.0	224.5	2.4–2.8	2.6
	Late Carboniferous monzonitic granite	1183.5–25,096.5	5880.8	42.0–772.0	431.8	2.4–2.7	2.6
	Late Carboniferous Syenogranite	343.7–33,232.7	5921.8	20.0–480.0	223.0	2.4–2.6	2.6
	Late Carboniferousgranitic mylonite	1922.9–8214.9	4090.3	4.0–299.0	35.4	2.3–2.7	2.5
	Middle Ordovician diorite	436.3–20,533.5	6118.2	27.0–2564.0	1031.2	2.5–2.9	2.7
Stratum	Ganhe Formation basalt	83.5–57,754.7	5773.6	387.0–2059.0	865.0	2.1–2.7	2.6
	Guanghua Formation rhyolite	888.5–4826.0	2857.3	13.0–554.0	145.6	2.1–2.7	2.5
	Jiufengshan Formation intermediate-acid tuff	118.4–5877.9	1485.7	40.0–235.0	100.6	2.3–2.9	2.6
	Longjiang Formation andesite	795.7–3745.7	1875.7	–	–	2.08–2.63	2.4
	Luohe Formation sandstone	706.5–31,699.2	5400.3	12.0–481.0	88.2	2.2–2.6	2.4

Intrusive rocks generally exhibit high resistivity values^37–39^. Notably, the Middle Ordovician diorite shows a wide resistivity range from 436.3 to 20,533.5 Ω m. The Late Carboniferous granitic mylonite has an average resistivity of 4090.3 Ω m, while the Late Carboniferous monzonitic granite and syenite granite demonstrate similarly high resistivity anomalies. The resistivity of Middle Jurassic granodiorite varies between 3435.4 and 14,977.9 Ω m, averaging at 5178.4 Ω m. Sandstones from the Luohe Formation strata also present high resistivity anomalies, ranging from 706.5 to 31,699.2 Ω m.

In contrast, volcanic rocks like the andesite of the Longjiang Formation, the intermediate-acid tuff of the Jiufengshan Formation, and the rhyolite of the Guanghua Formation display medium–low resistive anomalies with average resistivities of 1875.7 Ω m, 1485.7 Ω m, and 2857.3 Ω m, respectively. However, the Ganhe Formation basalt shows a high resistivity anomaly, averaging at 5773.6 Ω m.

Regarding magnetic susceptibility, the Middle Ordovician diorites have the strongest magnetic susceptibility with an average of 1031.2 × 10^–5^ SI, followed by the Late Carboniferous monzonitic granites at 431.8 × 10^–5^ SI. The Late Carboniferous granitic mylonites show the weakest magnetic susceptibility at 35.4 × 10^–5^ SI. Both the Middle Jurassic granodiorites and the Late Carboniferous syenogranites exhibit similarly low magnetic susceptibility rates, averaging around 200 × 10^–5^ SI.

Among the stratigraphic layers, the Luohe Formation sandstones have low magnetic susceptibility rates, while the intermediate-acid tuff of the Jiufengshan Formation and the rhyolite of the Guanghua Formation display weak magnetic susceptibility anomalies. The Ganhe Formation basalt has the highest magnetic susceptibility rate, varying from 387.0 to 2059.0 × 10^–5^ SI.

Density measurements indicate medium to low-density anomaly characteristics for the rocks. The Middle Ordovician diorites are the densest among the intrusive rocks with an average density of 2.7 × 10^3^ kg/m^3^, whereas the andesites of the Longjiang Formation have the lowest density at 2.4 × 10^3^ kg/m^3^. The sandstone of the Luohe Formation also has a low density, similar to the Longjiang Formation.

The significant variations in electrical properties, magnetic susceptibility, and density among the main rocks and stratigraphic layers provide a crucial basis for identifying strata and rock bodies^40^. These findings suggest that the region possesses the necessary physical properties for effective gravimetric, magnetic, and electrical exploration.

### Regional gravity anomaly characteristics

**Fig. 3:**
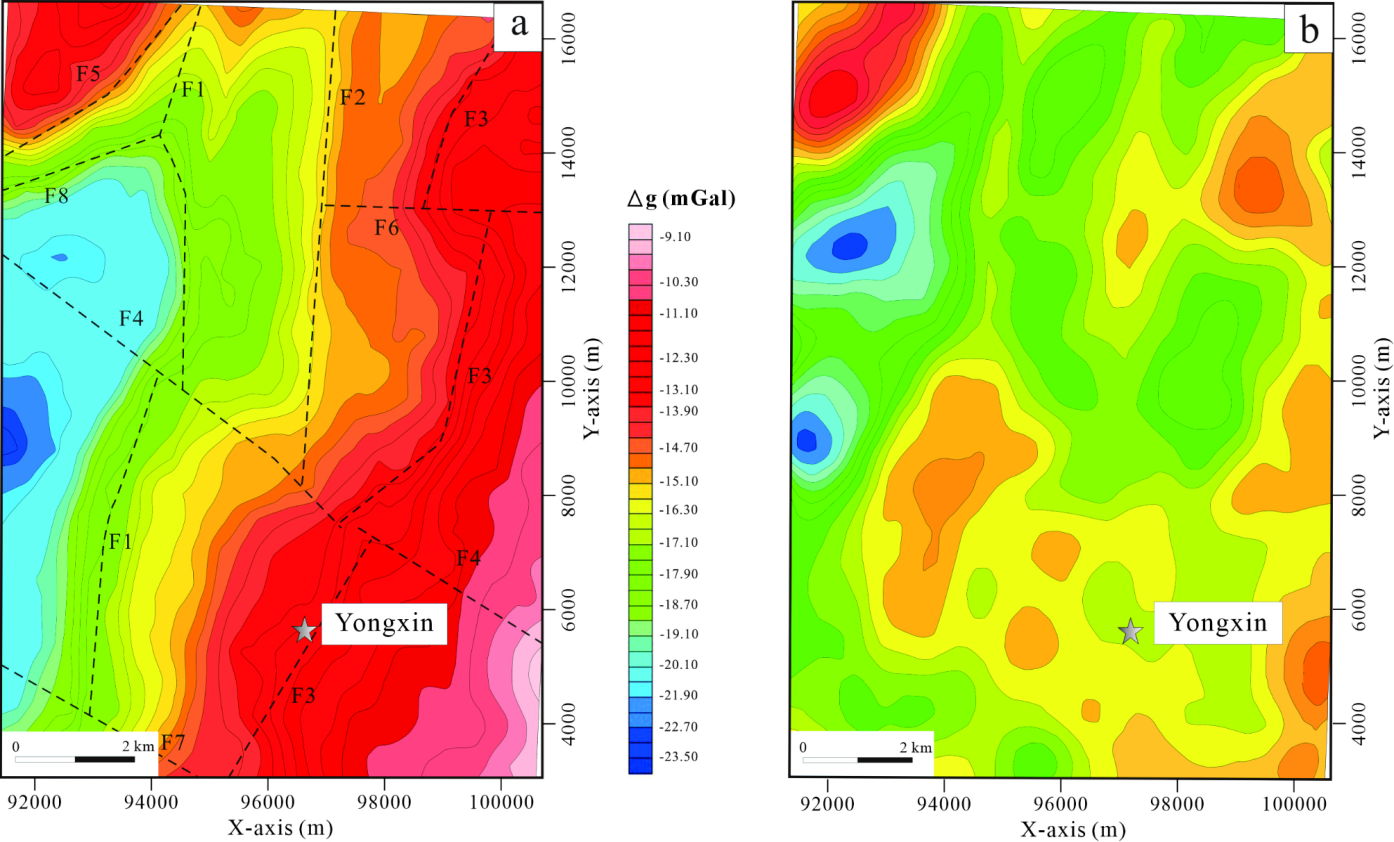
Gravity anomaly map. (**a**) Bouguer gravity anomaly map. (**b**) Residual gravity anomaly map. In the figure, the different faults are denoted by the letter “F”. (The figure is generated using RGIS (https://www.cgs.gov.cn/ddztt/kydh/2016kydh/Sbzs/201609/t20160923_405162.html) by Jun Chen).

The gravity anomalies observed in the study area typically manifest as higher values in the southeast and lower values in the west, with a range of intensities spanning from -32 to -2 × 10^–5^ m/s^2^. These anomalies predominantly align in a northwest direction, echoing the geological structural patterns prevalent in the entire region. Through rigorous analysis and interpretation of various datasets, including isogonic line distributions and gravity field attributes (Fig. 3a), we have inferred the existence of eight faults. Within the local gravity field, four distinct high anomalies (labeled G1-4) and three low anomalies (labeled L1-3) have been identified. Collectively, these anomalies display irregular and discontinuous patterns of distortion, underscoring the intricate and complex nature of the crustal structure within the study area (Fig. 3b).

### Regional magnetic anomaly characteristics

The magnetic field strength in the entire region typically falls within the range of -500 to 1500 nT. In the western part, the magnetic field exhibits a distinctive pattern of intermittent positive anomalies superimposed on a generally low and steady negative background. Prominently, there are island-like single or multi-peak positive anomalies of significant magnitude, predominantly aligned in a northeast direction. These anomalies are often associated with magnetic field values ranging from -500 to -1500 nT, which correspond to the underlying geology of Cenozoic Xishan basalt, Mesozoic Ganhe Formation, and Jiufengshan Formation volcanic rocks, primarily attributed to the basaltic rocks of the Ganhe Formation.

**Fig. 4:**
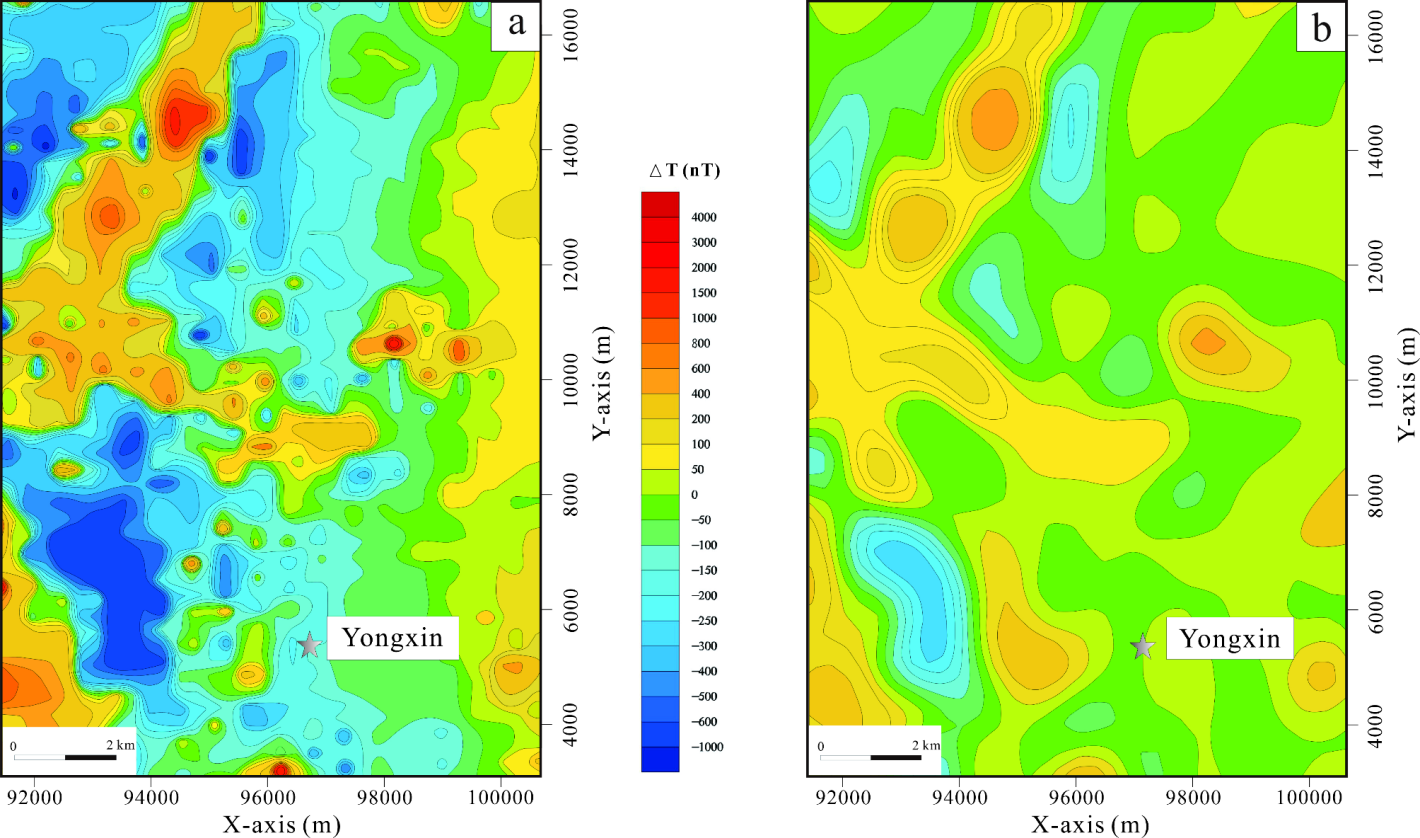
Magnetic anomaly contour plan. (**a**) Regional magnetic anomaly contour plan. (**b**) local magnetic anomaly contour plan. (The figure is generated using RGIS (https://www.cgs.gov.cn/Ddz.tt/kydh/2016kydh/Sbzs/201609/t20160923_405162.html) by Jun Chen).

In contrast, the magnetic field in the eastern region exhibits a more gradual variation, with strengths fluctuating between -50 and 50 nT. This subtle magnetic signature corresponds to geological features such as Late Carboniferous granitic mylonite and monzonitic granite (Fig. 4a). Notably, a majority of the mineralization occurrences discovered within the study area are situated in zones characterized by magnetic anomaly gradients or regions of reduced magnetic intensity. These mineralization points typically exhibit magnetic anomaly values within the range of -200 to 500 nT (Fig. 4b).

### Integrated geological features of AMT profiles

**Fig. 5:**
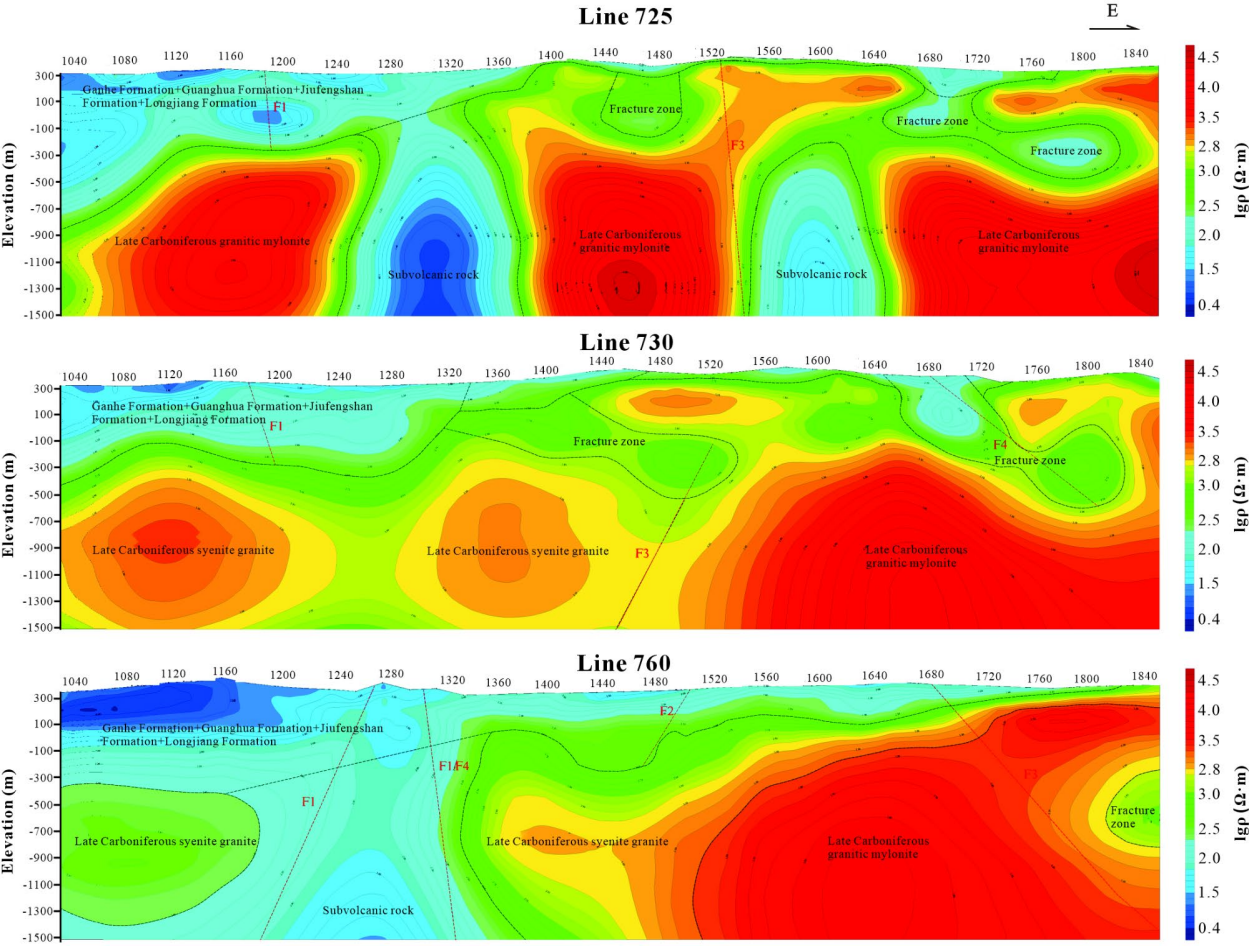
AMT resistivity cross-sectional diagram and inferred interpretation diagram of Line 725, Line 730 and Line 760. In the figure, the different faults are denoted by the letter “F”. The top axis of the middle plot represents the measurement point number. (The figure is generated using RGIS (https://www.cgs.gov.cn/Ddztt/kydh/2016kydh/Sbzs/201609/t20160923_405162.html) by Jun Chen).

This paper presents an integrated interpretation and analysis of inversion results from three representative AMT profiles, including Lines 725, 730, and 760 (Fig. 5). We chose the TE&TM inversion mode, which can effectively reduce the influence of 3D distortion, better distinguish the strata, and identify the spatial distribution characteristics of fault structures. Given the relative nature of high and low resistivity, a threshold of 500 Ω m is set as the dividing line between the two, based on statistical data from the work area’s rock electrical properties and prior experience.

Line 725 traverses the southern portion of the study area. The resistivity profile indicates that, apart from the section between points 1800 and 1860, all other areas near the surface exhibit layers of varying low resistivity, ranging from 20 to 500 Ω m. By correlating geological maps and available data, it is deduced that these near-surface low-resistivity layers are primarily attributed to low flood plain accumulations, weathered rock crusts, and strata from the Ganhe and Jiufengshan Formations. In the profile of Line 725, a distinct contrast between high and low resistivities is observed in the middle to lower sections, delineated by a clear boundary. Notably, the high-resistivity layer is segmented into three parts by two distinct low-resistivity zones. Specifically, points 1260 to 1360 and 1560 to 1640 mark two vertical low-resistivity areas. The former zone extends to the surface, likely due to subvolcanic rocks from the Ganhe Formation. The latter zone, however, remains subterranean, with localized high resistivity appearing above. This suggests that the low-resistivity area is caused by subvolcanic rocks, while the high resistivity above may result from fractured granitic mylonite within the high-resistivity layer (Fig. 5).

Line 730, also located in the southern part of the study area, exhibits predominantly low resistivity near the surface. The absence of this layer between points 1760 and 1820 is hypothesized to be due to a thin weathered crust exposing the underlying granitic mylonite. At a depth corresponding to point 1480, a gold ore body is exposed at the contact between syenogranite and granitic mylonite. Another gold ore body is visible in the low-resistivity zone at point 1670. Given that gold alone does not produce a low-resistivity response unless accompanied by significant amounts of other polymetallic minerals, combined with drilling and geological data, it is postulated that the observed low resistivity is primarily attributed to the weathered crust. Consequently, areas within and along the edges of high-resistivity layers, where low-resistivity zones and trends emerge, are identified as prime targets for future mineral exploration (Fig. 5).

Line 760 is situated in the heart of the study area, exhibiting a distinct resistivity gradient that increases from west to east. Between points 1020 and 1320, a low-resistivity layer is evident in the western segment. This layer is postulated to arise from the thicker sedimentary strata belonging to the Ganhe and Guanghua Formations close to the surface. Deeper down, it is conjectured that the Luohe Formation strata contribute to this low resistivity. A notable high-resistivity trend (ranging from 352 to 398 Ω m) emerges in the lower strata of the 1020 to 1120 cross-section, likely attributable to the presence of syenogranite. Furthermore, the exceptionally high resistivity observed between points 1440 and 1840 (spanning from 6310 to 10,000 Ω m) is believed to stem from granitic mylonite. In the middle of the farthest eastern section, a semicircular area displaying a low-resistivity trend is thought to result from rock disintegration (Fig. 5).

Drawing from the electrical traits exhibited across these three profiles, we can deduce the following from the AMT data in this region: The low-resistivity layer close to the surface likely originates from deposits associated with low-lying river floodplains, weathered rock crust, as well as strata from the Ganhe Formation, Longjiang Formation, Jiu Fengshan Formation, and other Mesozoic volcanic layers. On the other hand, the high-resistivity zones are primarily made up of syenogranite and granitic mylonite. The interior sections of these zones exhibit medium to low resistivity, indicating the possible existence of magmatic vents.

## Discussion

### The joint inversion of gravity, magnetic, and electrical data

The joint geophysical inversion method utilizes multiple physical parameters and various types of geophysical data for mutual constraint, which can effectively improve the accuracy of inversion results^41–46^. Utilizing the powerful inversion simulation capabilities of the RGIS software, we imported the actual exploration data to generate measured lines. By incorporating constraints such as geological conditions, rock physical properties, and drilling data, we continuously adjusted fault locations, rock mass ranges, and shapes to optimize the AMT initial model. These zoned models result from an interactive 2D modelling of gravity and magnetic data constrained by the two-dimensional distribution of the electrical resistivity obtained from the 2D inversion of AMT data. This optimization process aimed to achieve the best fit between the theoretical anomalies generated by the model and the measured anomalies, minimizing the mean square error of the simulation results. During the fitting process, we developed a clear understanding of the structural development, lithology, and stratigraphic conditions of the study area. This approach ensured that we did not overly pursue fitting to the observed data, preventing the loss of fundamental geological significance in the obtained model.This iterative optimization of the initial model ensures optimal parameter fitting for both theoretical and measured anomalies, while the mean square error of the simulation results meets our target requirements^47^.

This study conducted a gravity survey spanning 100 km^2^ and high-magnetic profile measurements along lines 730, 765, and 795 (Fig. 2). Given the significant spacing between profile lines, the study primarily relied on profile interpretations, using the plane anomalies only as a reference.

During the inversion process, the majority of the magnetic profile measurements were obtained in volcanic rock regions where magnetic field fluctuations are pronounced, posing challenges for quantitative inversion^48–51^. To enhance the inversion’s accuracy and quality, this study incorporated constraints from the bedrock depth and undulating features deduced from the AMT profiles. A joint gravity-magnetic and electrical inversion interpretation was performed for the three high-magnetic profile lines, including those with AMT profiles.

For line 730, the eastern section exhibited a higher magnetic field compared to the western section, with a consistently positive magnetic field in the east and an opposing field in the west. The geological map attributed this to Carboniferous granite in the east and the Mesozoic Cretaceous Jiufengshan Formation in the west. The gravity field along this line followed a similar trend, with higher values in the east and lower in the west. A prominent gravity bulge was observed around the 1190-point mark in the west.

**Fig. 6:**
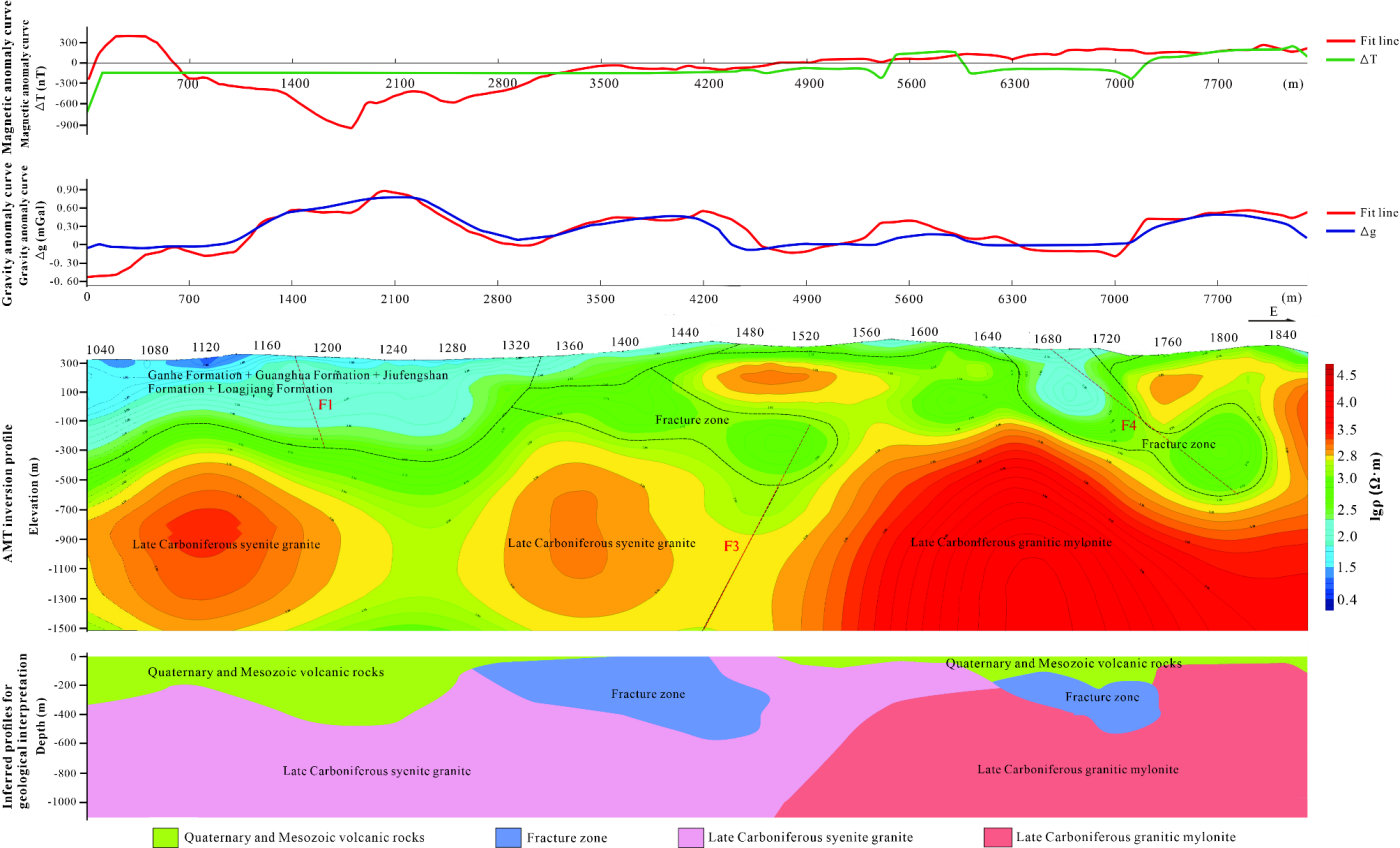
The joint inversion of gravity, magnetic and electrical information of Line 730. In the figure, the different faults are denoted by the letter “F”. The top axis of the middle plot represents the measurement point number. (The figure is generated using RGIS (https://www.cgs.gov.cn/Ddztt/kydh/2016kydh/Sbzs/201609/t20160923_405162.html) by Jun Chen).

Inversion results revealed that the profile’s lower section comprises granite, specifically granitic mylonite in the east and Syenogranite in the west. A thin layer of the Jiufengshan Formation and Quaternary cover rests above the orthoclase granite in the west, while the granitic mylonite in the east is predominantly exposed at the surface (Fig. 6).

By analyzing the anomalous characteristics of various gravity and magnetic profiles, along with electrical sounding profiles, the inversion results were extrapolated across the entire study area. This comprehensive approach facilitated the inference of the complete stratigraphic distribution within the study region.

**Fig. 7:**
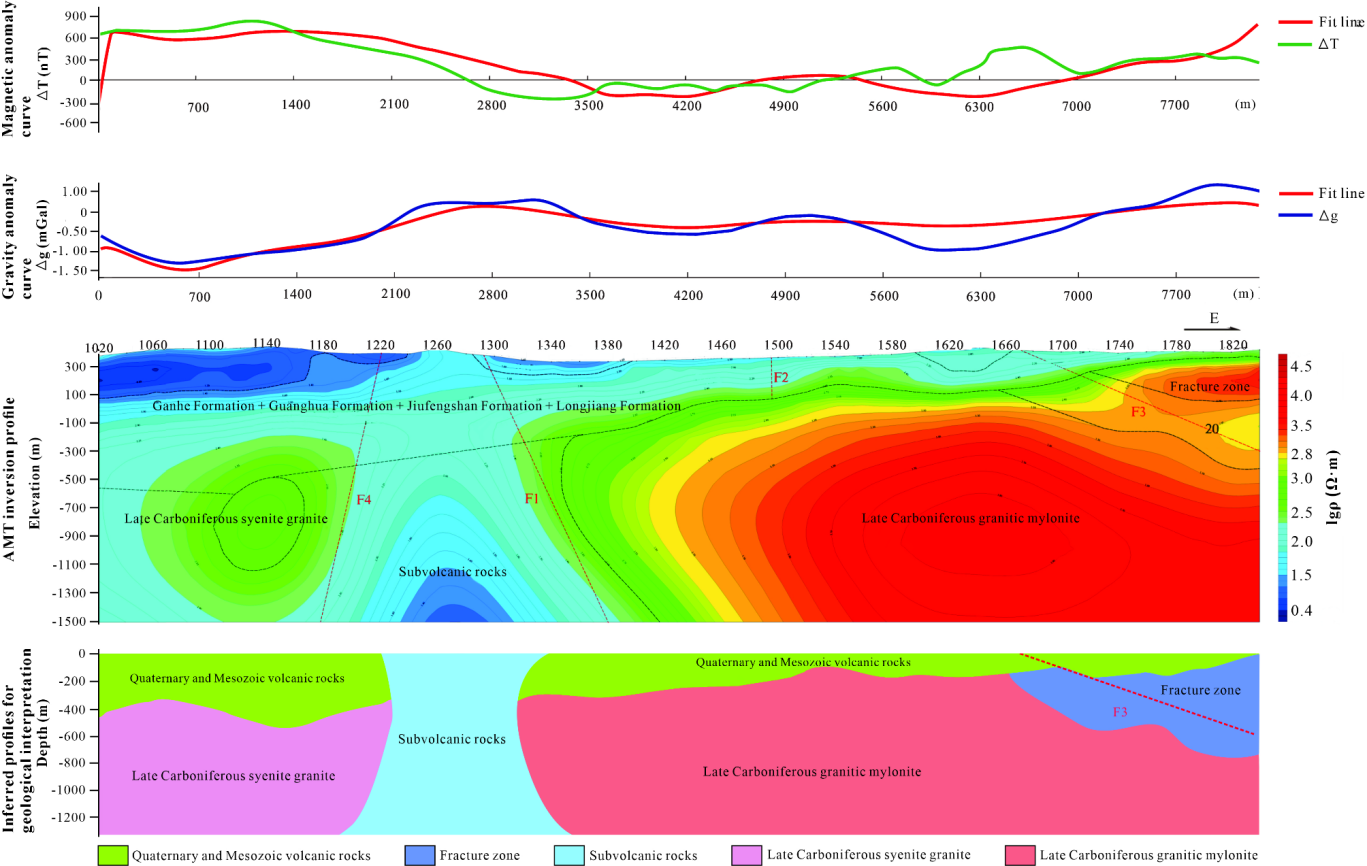
The joint inversion of gravity, magnetic and electrical information of Line 765. In the figure, the different faults are denoted by the letter “F”. The top axis of the middle plot represents the measurement point number. (The figure is generated using RGIS (https://www.cgs.gov.cn/Ddztt/kydh/2016kydh/Sbzs/201609/t20160923_405162.html) by Jun Chen).

Line 765, akin to line 730, displays a magnetic field that is stronger in the west and weaker in the east. The western magnetic field shows mild variations and local sawtooth patterns, mostly positive. The gravity and magnetic inversion section reveals that the syenite granite lies somewhat deeper, and the Quaternary and Mesozoic volcanic rock cover has thickened, reaching a maximum depth of approximately 500 m in the west. A notable difference from line 730 is that line 765 features a low magnetic value depression and a prominent high gravity value bulge between points 1220 and 1300 in the west. This corresponds to the subvolcanic rocks indicated on the geological map (Fig. 7).

**Fig. 8:**
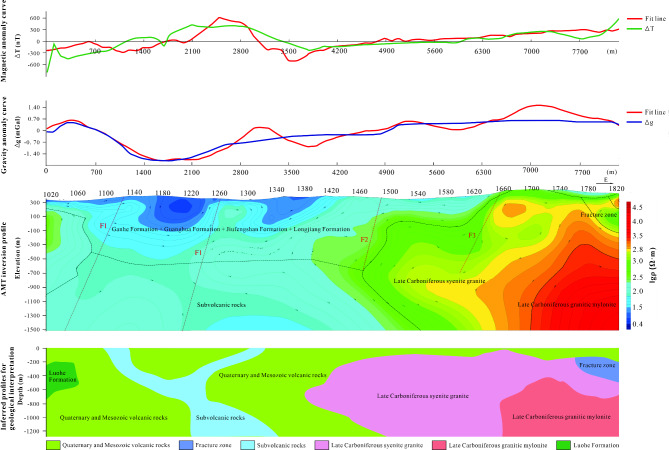
The joint inversion of gravity, magnetic and electrical information of Line 795. In the figure, the different faults are denoted by the letter “F”. The top axis of the middle plot represents the measurement point number. (The figure is generated using RGIS (https://www.cgs.gov.cn/Ddztt/kydh/2016kydh/Sbzs/201609/t20160923_405162.html) by Jun Chen).

In comparison to line 765, the cover in the eastern section of line 795 has increased in thickness. The western part of the line showcases the emergence of the Luohe Formation and subvolcanic rocks, where the Luohe Formation appears in the form of xenoliths. The magnetic field is predominantly negative in the west, while it tends to flatten out in the east. There is a significant gravity dip from points 1140 to 1220, which aligns with sedimentary rock strata. From point 1260 eastward, gravity progressively rises, correlating with intrusive rocks (Fig. 8).

Liang et al. speculated in their halo geochemistry study that further prospecting efforts should focus on the northwest of the Yongxin gold mine^52^. While Li et al. concluded that the deep regions of the northwestern area under study possess mineralization potential^53^. In light of these findings, this paper integrates gravity, magnetic, and electrical data interpretation with the geological context of the study area to derive a comprehensive understanding of the geological-geophysical features of the entire region.

The Late Carboniferous Syenogranite primarily emerges in the central-western and northeastern sections of the mining area, displaying a general northeast orientation and an irregular stock-like appearance. The upper portions of this rock formation are concealed by volcanic rocks belonging to the Guanghua Formation, and the western segment is intruded by Late Carboniferous granitic mylonite. Fracture zones are prevalent throughout the area, typically extending in a northeasterly direction, resulting in a zonal spatial distribution pattern characterized by a discontinuous block-like arrangement of subducted volcanic rocks.

Additionally, the Upper Ordovician Luhe Formation predominantly occurs in the northeastern segment of the study region, exhibiting a generally transgressive rhythmic pattern from its base towards the top. The base of this formation conforms to the adjacent Duobaoshan Formation, while its upper layers are overlaid by Mesozoic volcanic rocks, specifically comprising the Lower Cretaceous Ganhe Formation, Guanghua Formation, and Jiufengshan Formation.

By synthesizing geological and geophysical data, this study provides valuable insights into the complex geological setting encompassing multiple rock formations, fracture zones, and intrusive events, all of which hold significance in terms of the mineralization potential identified by preceding research.

Simultaneously, the distribution of fracture structures within the study area has undergone thorough inference and analysis, revealing the presence of eight fractures. These fractures primarily align in northwest, northeast, north–south, and near east–west directions. Notably, the northeast and northwest fractures constitute two regional, deep, and extensive fracture structures. The northeast fractures appear sparse and gently undulating, while the northwest fractures generally exhibit characteristics of tensile (torsional) fracture structures.

Drawing from the aforementioned interpretive results, a geological-geophysical model has been established. This model underscores that the gravity, magnetic, and electrical anomalies observed at the contact zones between Late Carboniferous orthoclase granite and granitic mylonite align precisely with the locations of typical mineral deposits. This finding suggests that the integrated geophysical information constraint inversion method possesses a certain degree of referentiality and feasibility.

To further pinpoint specific exploration targets, the Creatar XModeling 3D geological modeling software has been employed to build a three-dimensional geological model of the study area (Fig. 8). This model achieves a transparency of deep geological bodies, providing an enhanced understanding of the subsurface geology and aiding in the identification of potential mineral resources.

### Analysis of favorable conditions for mineralization

**Fig. 9:**
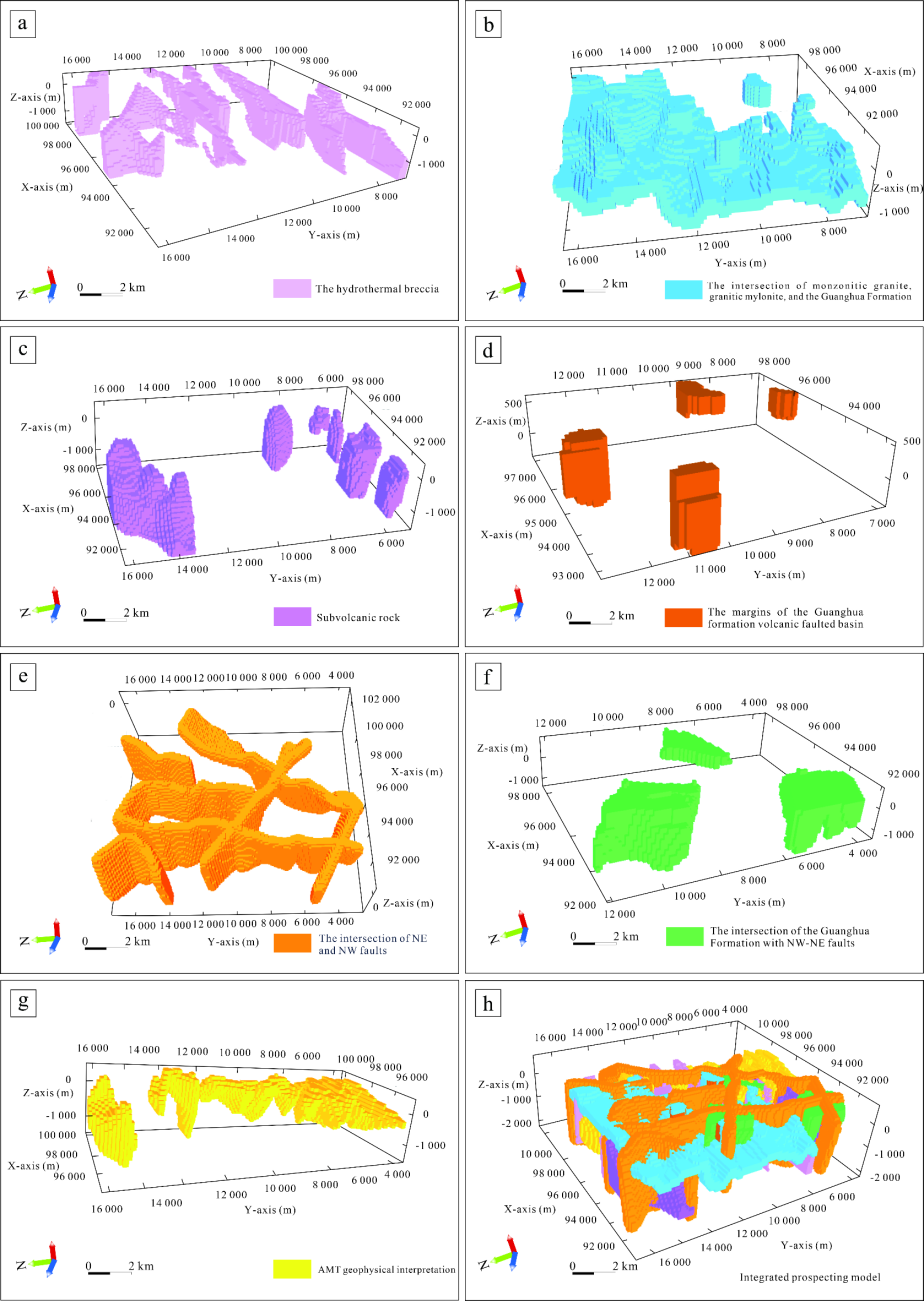
Favorable factors for mineralization and integrated prospecting model. (**a**) Hydrothermal breccia. (**b**) The intersection of monzonitic granite, granitic mylonite, and the Guanghua Formation. (**c**) Subvolcanic rock. (**d**) The margins of the Guanghua Formation volcanic faulted basin. (**e**) The intersection of NE and NW faults. (**f**) The intersection of the Guanghua Formation with NW-NE faults. (**g**) AMT geophysical interpretation. (**h**) Comprehensive mineral prospecting model. (The figure is generated using micromine (https://www.wiseminetech.com/) by Jun Chen).

This study conducted a comprehensive analysis of the geological-geophysical model (Fig. 9) of the Yongxin gold mining area, identifying key favorable conditions (Table 2) for mineralization and constructing an integrated ore-finding model. We combined a three-dimensional geological model with various geophysical methods, including gravity, magnetic, and electromagnetic surveys, to conduct a detailed assessment of the mineralization potential in the deep part of the mining area.

**Table 2:** Comprehensive prospecting model for the study area.

Types of mineral deposit	Favorable factors for mineralization	Feature variables
Epithermal gold deposit	Favorable geological features	The hydrothermal breccia (Fig. 9a)
		The intersection of monzonitic granite, granitic mylonite, and the Guanghua Formation (Fig. 9b)
		Subvolcanic rock (Fig. 9c)
		The margins of the Guanghua formation volcanic faulted basin (Fig. 9d)
	Favorable mineralization structures	The intersection of NE and NW faults (Fig. 9e)
		The intersection of the Guanghua Formation with NW-NE faults (Fig. 9f)
	Favorable geophysical characteristics for mineralization	AMT geophysical interpretation (Fig. 9g)

1. *Geological conditions*. The geological-geophysical model of our study area reveals strata spanning the Cenozoic, Mesozoic, and Paleozoic eras. The Cenozoic and Mesozoic strata, including river alluvium and formations like Guanghua, Longjiang, Ganhe, and Jiufengshan, serve as cover layers^54,55^. The Paleozoic strata consist primarily of the Duobaoshan, Luohe, Niqiuhe, and Yaosangnan formations.

Significant geophysical anomalies are observed at the contact zones between Late Carboniferous syenogranite and granitic mylonite, specifically within the Mesozoic Guanghua formation. These anomalies suggest that the hydrothermal breccia (Fig. 9a) located around these contacts is a potential ore body site. Furthermore, the intersection of these rock types emerges as a promising mineralization zone.

In addition, the Yongxin gold deposit lies at the periphery of an Early Cretaceous volcanic basin^56^, characterized by vast exposures of volcanic and subvolcanic rocks. Gold mineralization is spatially associated with subvolcanic masses and the marginal zones of this volcanic basin (Fig. 9b, c, d), indicating a close relationship between volcanism and gold mineralization.

(2) *Tectonic conditions*. The area features faults oriented northwest, northeast, north–south, and nearly east-west^57^. Deep fault structures in the northeast and northwest (Fig. 9e) significantly influence mineralization, magmatic activities, and mineralization processes. The northeast-oriented faults are more prominent and characterized by compressive fracture tectonics, while the northwest-oriented faults are smaller and typically exhibit tensile fracture characteristics.

A higher number of gold and polymetallic ores are primarily associated with the Guanghua Formation and these faults (Fig. 9f). Mineralization anomalies and hydrothermal alteration sites have developed at their intersections, indicating a close spatial correlation between gold deposits and these tectonic structures.

(3) *Geophysical conditions and integrated prospecting model*. Seven gravity anomalies were identified through gravity and magnetic surveys. These anomalies primarily occur at the contact zone between Mesozoic volcanic-subvolcanic rocks and Late Carboniferous granodioritic cherts, observable in gradient zones or regions of low magnetic intensity.

AMT measurements reveal low rock resistivity values, corresponding to a mid-to-low resistivity gradient zone (Fig. 9g). Previous studies and our findings suggest that the mineralization site of the Yongxin gold deposit is located at a specific contact zone.

Considering all factors, we postulate that magnetic anomaly gradient zones or low magnetic field regions, combined with mid-to-low resistivity gradient zones, constitute favorable locations for mineralization. Fracture zones aligned with low-resistivity strata visible in AMT images are believed to be closely associated with mineralization processes.

Utilizing a comprehensive mineral prospecting model (Fig. 9h), we discovered that the known Yongxin gold ore body is situated within and adjacent to a hydrothermal breccia body at a specific contact zone. This integrated approach provides valuable insights for future mineral exploration efforts in the study area.

### Mineralization prediction

**Fig. 10:**
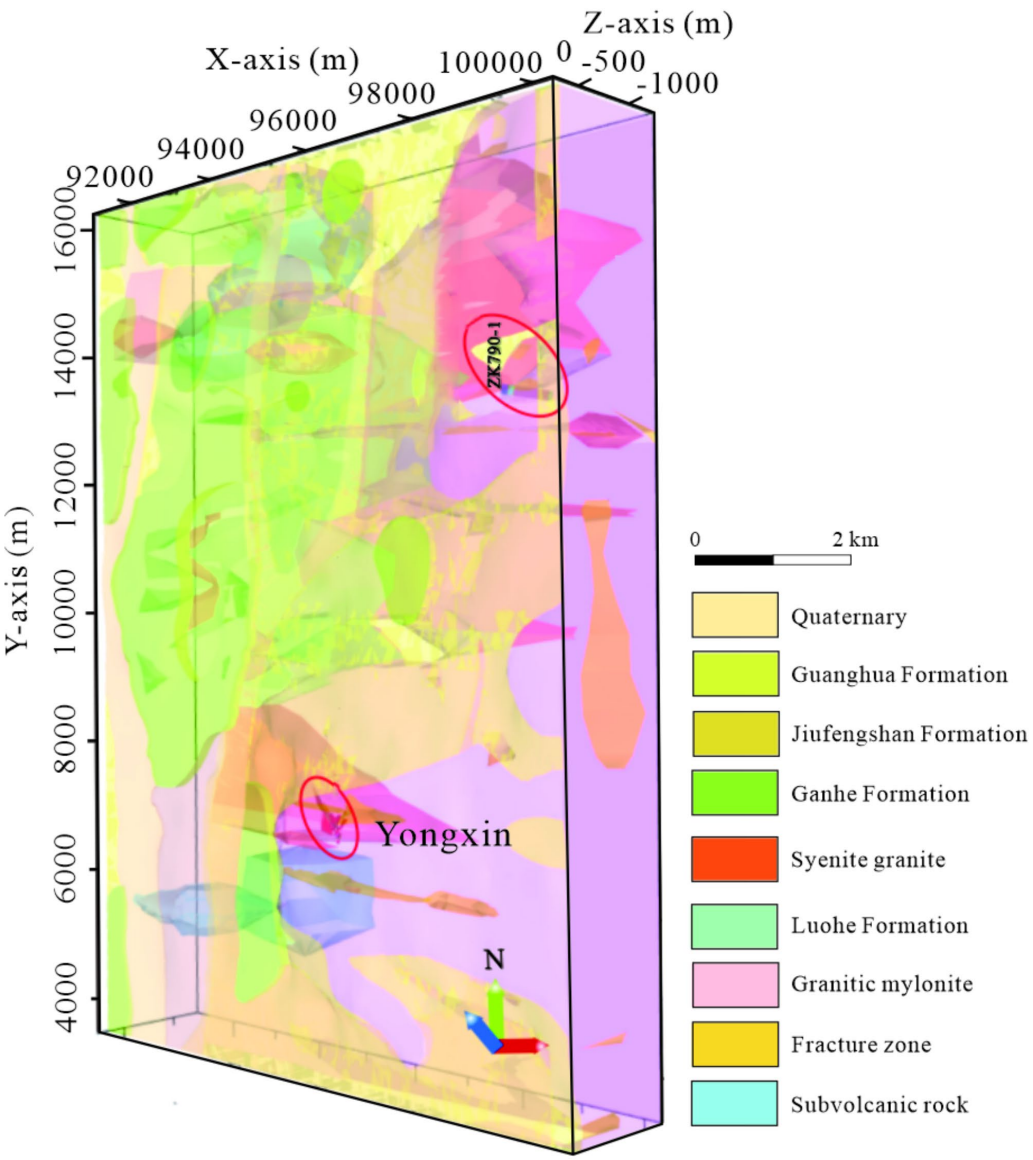
3D geological model and drilling verification location map. (The figure is generated using micromine (https://www.wiseminetech.com/) by Jun Chen).

The ore body corresponds to the favorable mineralization zone identified through the integrated interpretation of gravity, magnetism, and electrical anomalies. Additionally, the fracture zone and the junction of rock masses located northeast of the study area bear geological and geophysical resemblances to the known Yongxin gold ore body, implying a comparable mineralization context (Fig. 10). The hydrothermal breccia body, situated at the boundary between Late Carboniferous granitic mylonite and syenigranite, exhibits geophysical markers of a medium-to-low resistive gradient zone, suggesting potential mineralization at deeper levels.

To investigate this prospect, drilling hole ZK790-1 was selected for exploration. Drilling outcomes disclosed that the deeper borehole segments predominantly consist of strongly silicified granodioritic mylonite, with intermittent occurrences of diorite-porphyrite and quartz veins. Notably, multiple mineralization layers of varying thicknesses were encountered. The concordance between the encountered rock types and thicknesses with our interpreted results underscores the efficacy of integrated geophysical interpretation techniques in metal mineral resource exploration.

## Conclusions

1. The Yongxin gold deposit is primarily controlled by north-east trending faults and is hosted within the contact zone between Late Carboniferous granitic mylonite and syenogranite. Our study precisely locates the ore bodies within the high-to-low resistivity gradient zone.
2. This research innovatively integrates audio-frequency magnetotelluric (AMT) sounding, gravity surveys, and high-magnetic profiling, leveraging 3D modeling techniques to effectively identify lithological variations and deep ore-forming geological features within the study area. This approach demonstrates a highly efficient and comprehensive interpretation method.
3. By constructing a 3D geological model, we have achieved “transparency” of the geological structure within a 1.5 km depth range in the study area. Combining ore-forming geological conditions with geophysical interpretation results, we have summarized the favorable mineralization conditions of the Yongxin gold mining area and developed a comprehensive ore-prospecting model. Drilling verification has successfully identified multiple mineralized zones at depth, confirming the synergy between integrated geophysical exploration and 3D modeling, and highlighting their significant potential and application prospects in metal mineral exploration.

## Data Availability

The datasets generated and/or analysed during the current study are not publicly available due [REGULATORY REQUIREMENTS FOR CONFIDENTIAL DATA] but non confidential data are available from the corresponding author on reasonable request.
